# Molecular Pharmacology of Inflammation Resolution in Atherosclerosis

**DOI:** 10.3390/ijms23094808

**Published:** 2022-04-27

**Authors:** Stanislav Kotlyarov, Anna Kotlyarova

**Affiliations:** 1 Department of Nursing, Ryazan State Medical University, 390026 Ryazan, Russia; 2 Department of Pharmacology and Pharmacy, Ryazan State Medical University, 390026 Ryazan, Russia; kaa.rz@yandex.ru

**Keywords:** atherosclerosis, inflammation, innate immunity, lipids, specialized pro-resolving mediators, pharmacology

## Abstract

Atherosclerosis is one of the most important problems of modern medicine as it is the leading cause of hospitalizations, disability, and mortality. The key role in the development and progression of atherosclerosis is the imbalance between the activation of inflammation in the vascular wall and the mechanisms of its control. The resolution of inflammation is the most important physiological mechanism that is impaired in atherosclerosis. The resolution of inflammation has complex, not fully known mechanisms, in which lipid mediators derived from polyunsaturated fatty acids (PUFAs) play an important role. Specialized pro-resolving mediators (SPMs) represent a group of substances that carry out inflammation resolution and may play an important role in the pathogenesis of atherosclerosis. SPMs include lipoxins, resolvins, maresins, and protectins, which are formed from PUFAs and regulate many processes related to the active resolution of inflammation. Given the physiological importance of these substances, studies examining the possibility of pharmacological effects on inflammation resolution are of interest.

## 1. Introduction

Atherosclerosis is a global problem of modern society [1]. The magnitude of its prevalence and the significance of the clinical consequences, to which the progression of atherosclerosis leads, emphasize the need to search for new methods of its prevention and treatment [2]. Such diseases as coronary heart disease, stroke, and peripheral arterial disease make a significant contribution to the morbidity and mortality of the population [3,4]. The importance of the problem is reinforced by the fact that patients with atherosclerosis often have one or more comorbid diseases, which can worsen the condition of the patient [5,6]. In addition, patients may not seek medical care for a long time but do so only at clinically pronounced stages, which correspond to significant morphological changes in the vessels, which is an important obstacle to effective treatment and negatively affects the prognosis.

Despite a significant increase in the number of studies on the pathophysiology of atherosclerosis, many aspects of its initiation and progression are still unknown. The clinical and experimental data suggest a multifaceted role of lipids in atherogenesis [7,8]. The infiltrative theory of atherogenesis allowed us to emphasize the importance of dietary fats and dyslipidemia in enhancing lipid accumulation in the vascular wall [9,10]. Further studies have shown that the importance of lipids is not limited to passive participation as a substrate for the formation of the morphological basis of atherosclerosis. A growing body of evidence suggests that atherosclerosis is characterized by an imbalance between inflammation and the inflammation control mechanisms [8]. Lipids have demonstrated diverse roles in the regulation of inflammation in the vascular wall [11,12]. The information obtained in recent years has broadened the understanding of the role of lipid mediators of inflammation in atherogenesis, which allows us to pay attention to their therapeutic potential [13,14]. 

Medication regulation of inflammation activity in the vascular wall in atherosclerosis is a promising strategy. Moreover, in addition to affecting the inflammation activation phase, it seems important to regulate inflammation resolution, which can improve the balance between these two phases of inflammation and positively influence the clinical course of atherosclerosis. In this regard, the purpose of this review is to discuss the mechanisms of inflammation resolution in atherosclerosis involving lipid mediators, as well as the prospects for pharmacological effects on them.

## 2. Involvement of Lipid Mediators in Atherogenesis

**Figure 1:**
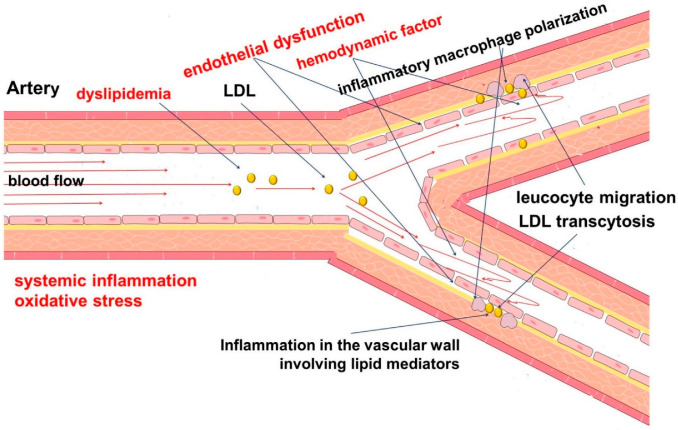
Pathophysiology of atherosclerosis. The development of atherosclerosis involves a complex chain of events, the initiating step of which is considered to be local hemodynamic disorders (turbulent blood flow), dyslipidemia, systemic inflammation, and oxidative stress. Endothelial dysfunction contributes to increased permeability to cells and lipids, with their subsequent accumulation in the vascular wall. This leads to the development and maintenance of inflammation.

The development of atherosclerosis begins with the accumulation of lipoproteins containing apolipoprotein B in the subendothelial intima of the arteries [15,16]. This is accompanied by a number of common factors, such as hyperlipidemia, oxidative stress, and systemic inflammation, as well as vascular factors, such as endothelial dysfunction and endothelial cell activation (Figure 1) [16,17,18].

Endothelial cells are key participants in atherogenesis [19,20]. They perform a number of important functions to ensure adequate hemodynamics and to actively respond to changes in blood flow patterns [21]. The local hemodynamic characteristics of blood flow are an important factor contributing to the development of atherosclerosis [22,23]. This corresponds to an increase in endothelial lipid transcytosis, which has several mechanisms [24]. In addition, vascular endothelial cells regulate immune processes in the vascular wall through the expression of adhesion molecules that regulate the recruitment of leukocytes to the lesion site [25].

Endothelial dysfunction plays an important role in the pathogenesis of atherosclerosis [26,27]. It is known that endothelium, which produces a number of biologically active substances, is involved in the regulation of hemodynamics and blood cell and vascular wall behavior, as well as lipid permeability [28,29,30]. Disorders of this function are associated with the accumulation of lipids and cells and the development of inflammation. The causes and mechanisms of endothelial dysfunction are the subject of numerous studies. Genetic predisposition may play an important role in the development of endothelial dysfunction and cardiovascular diseases [31]. In addition, dysregulation of the innate immune system, such as the complement system, may be involved in the pathogenesis of atherosclerosis [32].

Atherogenesis is characterized by the recruitment of monocytes to the endothelium, their migration into the vessel wall, and their differentiation into macrophages [33]. They can be polarized into different subtypes with different functions. When activated by interferon (IFN)-γ, tumor necrosis factor alpha (TNF-α), and Toll-like receptor (TLR) ligands such as lipopolysaccharide (LPS), macrophages are polarized into the pro-inflammatory M1 phenotype [34,35]. The M1 phenotype is associated with the production of pro-inflammatory cytokines and lipid mediators. In contrast, the alternative activation of macrophages (M2), when stimulated by interleukin-4 (IL-4) or IL-13, is associated with the resolution of inflammation and efferocytosis of apoptotic cells, which promotes tissue repair [36,37,38]. It should be noted that the division of macrophages only into the M1 and M2 phenotypes is a significant simplification of the real picture but seems convenient for a better interpretation of the processes taking place in an atherosclerotic plaque.

Both macrophage phenotypes are present in atherosclerotic plaques, with M1 macrophages predominating in plaque regions prone to rupture and M2 macrophages associated with stable plaques [39,40]. Within advanced plaques, M1 macrophages mostly localize near the lipid core, whereas M2 macrophages mostly cluster outside the lipid core [41]. M1 macrophages play a significant role in the progression of atherosclerosis, including monocyte recruitment, the maintenance of chronic inflammation, and the development of vulnerable plaques. In contrast, M2 macrophages are associated with decreased plaque inflammation and plaque regression [42,43]. 

The progression of atherosclerosis is associated with the uptake of lipoproteins by macrophages and their transformation into foam cells [44]. The lipid overload of macrophages and the uptake of oxidatively modified low-density lipoproteins (LDL) trigger pro-inflammatory responses [45]. The inflammatory activation of macrophages is also promoted by their cholesterol overload due to impaired reverse cholesterol transport involving ABCA1 and ABCG1 transporters [46]. In addition, other cells found in the area of the atherosclerotic lesion, including T cells and dendritic cells, also contribute to the increased expression of pro-inflammatory cytokines and eicosanoids, which support inflammation [18,47,48].

Inflammation is a universal mechanism that occurs in response to a variety of tissue injuries, both infectious and noninfectious. The innate immune system has multiple overlapping tools to initiate and maintain inflammation. Accumulated evidence suggests that the inflammation process not only has an initialization phase, but also an active resolution phase [49]. The resolution phase of inflammation is mediated by a number of biological factors and coordinates with the inflammation phase, which together play an important role in providing immune tissue homeostasis [50]. This coordination allows the organism to control inflammation in order to minimize tissue damage [51]. Some researchers suggest that there is also a “post-inflammatory” phase, which is also anti-inflammatory in its role. This phase is regulated by macrophages and dendritic cells. It is necessary for the coordination of the subsequent immune response through its influence on adaptive immunity [52,53,54].

**Figure 2:**
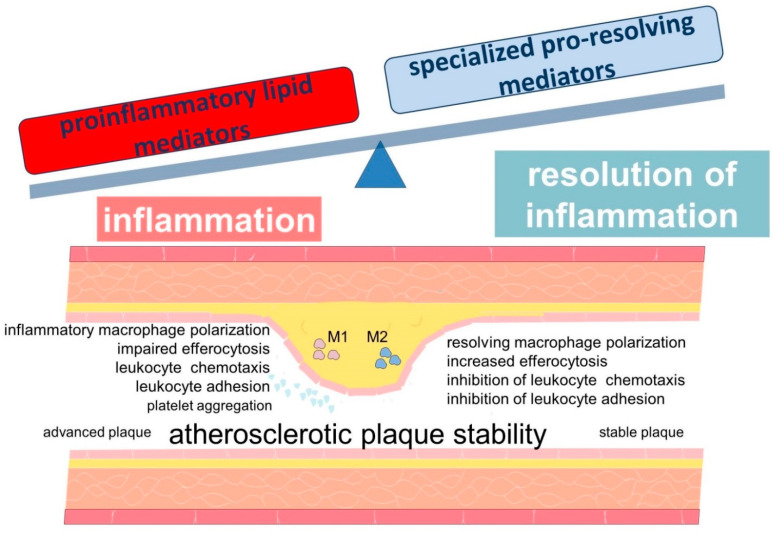
Scheme demonstrating the role of lipid mediators in the development of inflammation in atherosclerosis. The development and progression of atherosclerosis are associated with an imbalance between inflammation and inflammation resolution. Lipid mediators derived from PUFAs are involved in these processes.

Bioactive lipids, which are metabolites of fatty acids, play an important role in both maintaining and resolving inflammation [55]. They are involved in the regulation of multiple processes related to inflammation and may be actively involved in the pathogenesis of atherosclerosis [56,57]. Leukotrienes are considered to be key participants in inflammation in atherosclerosis. In turn, members of the family of lipid mediators, called “specialized pro-resolving mediators” (SPMs), play a key role in the active resolution of inflammation [58,59]. Atherosclerosis is characterized by an imbalance between the levels of pro-inflammatory and specialized pro-resolving mediators, resulting in persistent inflammation (Figure 2) [18,60].

SPMs are formed enzymatically from several polyunsaturated fatty acids (PUFAs) [61,62,63]. Lipoxins are formed from omega-6 arachidonic acid, while resolvins, protectins, and maresins are synthesized from omega-3 PUFAs such as eicosapentaenoic acid and docosahexaenoic acid [64]. Free fatty acids are a source of PUFAs for the synthesis of SPMs. Fatty acids for the formation of SPMs can also be released from membrane phospholipids using phospholipases [65,66]. 

Thus, PUFAs are a substrate for the formation of both pro- and anti-inflammatory lipid mediators. Plasma membrane phospholipids are considered to be a depot for PUFAs, which, after release, can be used for the synthesis of various lipid mediators of inflammation [67]. Given these data, it should be noted that numerous studies have evaluated the atheroprotective role of ω-3 PUFAs [68]. Their use is recommended as part of an antiatherogenic diet or as a medication, based on the results of the analysis of clinical data. It is important to note that discussions regarding the clinical efficacy of ω-3 PUFAs are ongoing and are the subject of a separate analysis [69].

## 3. Specialized Pro-Resolving Mediators

The regulation and control of inflammation is important for the maintenance of immunological homeostasis in tissues. The innate immune system has a variety of mechanisms with which to regulate inflammation. The production of eicosanoids in acute experimental inflammation is known to be time-coordinated. The first stage is the biosynthesis of pro-inflammatory leukotrienes (LT) and prostaglandins (PG), which is accompanied by the involvement of polymorphonuclear neutrophils (PMN). Then, there is a switch to the synthesis of lipoxins (LX), which have an anti-inflammatory effect. The switching of eicosanoid biosynthesis pathways from pro-inflammatory leukotriene B4 (LTB4) by 5-lipoxygenase (5-LOX) to lipoxin A4 (LXA4) by 15-LOX was shown to occur involving PMN exposed to PGE2 [70]. These data suggest that eicosanoids of the first phase of inflammation contribute to the switch to the biosynthesis of anti-inflammatory lipids [70]. Changes in individual classes of eicosanoids can have a significant impact on the duration of the inflammatory response.

It should be noted that the regulation of inflammation involving lipid mediators has complex control mechanisms. Indeed, 5-LOX is involved in the formation of both pro-inflammatory leukotrienes and pro-resolving lipoxins [71]. At the same time, the regulation of the enzymatic activity product depends on the subcellular localization of 5-LOX [18,72]. 5-LOX plays an important role in the formation of leukotrienes and a number of SPMs [72,73]. The increased expression of 5-LOX has been shown in human atherosclerotic lesions, with the expression levels correlating with signs of plaque instability in carotid arteries [74]. In addition, the 5-LOX mRNA levels were higher in patients with a history of recent clinical events, such as transient ischemic attack of the brain or minor stroke [74].

Another important factor is that the formation of SPMs involves the intercellular exchange of intermediate biochemical substances between endothelial cells, leukocytes, platelets, and vascular smooth muscle cells (VSMCs) [75]. At the same time, the detailed characterization of the cellular sources of SPM synthesis in the vascular wall remains a subject of debate.

Given the role of noninfectious inflammation in atherosclerosis, there is increasing evidence that atherogenesis is characterized by an imbalance between the production of pro-inflammatory lipid mediators and SPMs. The resulting effect of these disorders is an increase in leukocyte recruitment to the foci of atherosclerotic lesions, pro-inflammatory polarization of macrophages, and impaired efferocytosis [18]. In the further progression of atherosclerosis, the imbalance between the SPMs and pro-inflammatory leukotrienes contributes to the instability of atherosclerotic plaques. For example, SPMs, especially resolvin D1 (RvD1), and the ratio of SPMs to pro-inflammatory LTB4 have been shown to be significantly reduced in the vulnerable regions of human carotid atherosclerotic plaques [76]. Thus, the ratio of RvD1 to LTB4 correlated strongly with plaque severity [76]. Experimental data in fat-fed *Ldlr*^−/−^ mice also showed decreased SPMs in advanced plaques. However, the administration of RvD1 to these mice restores the RvD1:LTB4 ratio to the level of less developed lesions. This contributes to reduced oxidative stress, reduced necrosis, improved efferocytosis, and thickened fibrous cap, resulting in greater plaque stability [76]. 

Thus, the lipid mediators derived from PUFAs show different roles in inflammation and atherogenesis. For example, arachidonic acid, which is a metabolite for the synthesis of both pro- and anti-inflammatory mediators, is at the crossroads of inflammation pathways. Given that SPMs are an important mechanism of inflammation regulation and resolution, this makes these lipid mediators a significant participant in atherogenesis. [18,77].

### 3.1. Lipoxins

Lipoxins are the first identified class of SPMs. The chemical structure of lipoxins includes three hydroxyl residues and four double bonds. The origin from ω-6 arachidonic acid and the above structural features distinguish lipoxins from other SPMs, which are formed from ω-3 fatty acids.

**Figure 3:**
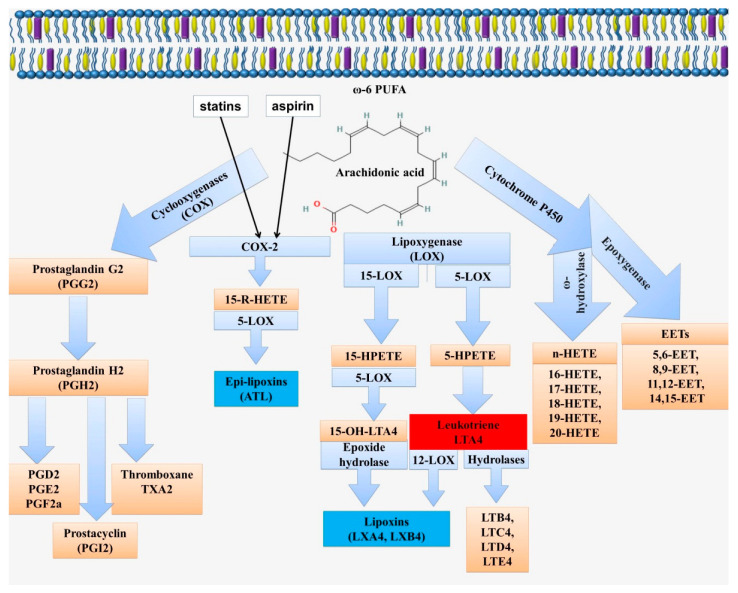
Scheme of biosynthesis of lipid mediators from arachidonic acid. Arachidonic acid can be metabolized via the cyclooxygenase (COX), lipoxygenase (LOX), or cytochrome P 450 pathways (CYP). Enzymatic conversion through the COX pathway leads to the formation of prostaglandins (PG). The LOX pathway is associated with the formation of lipoxins (LX) and leukotrienes (LT) via 5-LOX, 12-LOX, and 15-LOX. This pathway includes the formation of the intermediate metabolites 5-/15-hydroperoxyeicosatetraenoic acid (5-/15-HpETE) and 5-/15-hydroxyeicosatetraenoic acid (5-/15-HETE). The ω-hydroxylase activity of CYP enzymes leads to the formation of hydroxyeicosatetraenoic acids (16-, 17-, 18-, 19-, and 20-HETE). The epoxygenase activity of CYP enzymes is associated with the formation of arachidonic acid epoxides or epoxyeicosatrienoic acids (EETs; 5,6-EET, 8,9-EET, 11,12-EET and 14,15-EET), known as endothelial-derived hyperpolarizing factors.

Lipoxins are synthesized in two major pathways by the sequential action of lipoxygenase (LOX) enzymes, including 5-, 12-, and 15-LOX (Figure 3). The first pathway involves the enzymatic conversion of leukotriene A4 by 12-LOX [78]. The second pathway of lipoxin synthesis involves the action of 15-LOX and 5-LOX on arachidonic acid [79].

As previously noted, the subcellular localization of 5-LOX is at the intersection of pathways that determine the formation of pro-inflammatory leukotrienes or pro-resolving lipoxins. In particular, the nuclear localization of 5-LOX contributes to the biosynthesis of pro-inflammatory leukotriene [80]. This is because nuclear 5-LOX is located near the leukotriene A4 hydrolase, which leads to the conversion of arachidonic acid to leukotrienes (LTB4) [81,82,83]. The nuclear localization of 5-LOX is associated with its phosphorylation at Ser^271^ by MAPK-activated protein kinase 2 (MK2), which can be stimulated by MAPK p38 activation [18,80,82,83,84,85,86]. In contrast, the cytoplasmic localization of the unphosphorylated form of 5-LOX is associated with SPM formation. This is due to the proximity of 5-LOX to 12/15-LOX at the cytoplasmic localization, which promotes the conversion of LTA4 to LXA4.

It should be noted that 5-LOX activation is associated with five lipoxygenase activating protein (FLAP), which acts as an arachidonic acid transfer protein [87]. FLAP is a nuclear membrane protein and is required for the synthesis of both LT and LXA4/RvD1 [88]. Polymorphisms of the *ALOX5AP* gene encoding FLAP contribute to the risk of coronary heart disease in patients with familial hypercholesterolemia, as well as the development of myocardial infarction [89,90,91]. The FLAP antagonist BRP-201 causes a switch in the class of lipid mediators produced in human macrophages, shifting LT biosynthesis toward SPMs [92]. This may be related not only to FLAP inhibition but also to the stimulation of 15-LOX-1 activity in M2 macrophages [92].

Interestingly, however, the activation of MerTK in human macrophages, which is a macrophage receptor that mediates efferocytosis, leads to the ERK-mediated expression of sarcoplasmic/endoplasmic reticulum calcium ATPase 2 (SERCA2), which decreases cytosolic Ca^2+^ concentration [80]. This in turn suppressed Ca^2+^/calmodulin-dependent protein kinase II (CaMKII) activity and reduced MAPK p38 and MK2 kinase activity. The resulting effect is an increase in the amount of the unphosphorylated cytoplasmic form of 5-LOX and an increase in SPM formation [80]. Thus, MerTK signaling in macrophages promotes the production of 5-LOX-derived SPMs and contributes to the process of inflammation resolution [93]. This has important implications for the coordination of the different mechanisms involved in the resolution of inflammation.

Lipoxin A4 (LXA4 or (5S,6R,15S)-Trihydroxy-(7E,9E,11Z,13E)-eicosatetraenoic acid) and lipoxin B4 (LXB4 or (5S,6E,8Z,10E,12E,14R,15S)-5,14,15-Trihydroxy-6,8,10,12- icosatetraenoic acid) have currently been identified. In addition, their epimers, such as 15-epi-LXA4 and 15-epi-LXB4, are known. The formation of lipoxin epimers is associated with an aspirin-dependent pathway, which is an important therapeutic effect of this medicine (Figure 3) [94]. Aspirin modifies cyclooxygenase-2 (COX2) by acetylation in Ser^530^. This modification, limits the access of arachidonic acid to the catalytic core of COX-2 and promotes the switch from the production of PGH2, a prostaglandin precursor, to 15-R-HETE ((15R)-15-hydroxy-5,8,11-cis-13-trans-eicosatetraenoic acid), which is then converted by 5-LOX to 15-epi-LXA4 [95,96]. This pathway can be realized during intercellular interactions between leukocytes and endothelial cells [94]. In this case, the transcellular biosynthesis of aspirin-triggered lipoxins (ATLs) is carried out through the formation of 15R-HETE by the endothelium and its delivery to the adherent leukocytes [94,97]. In turn, 15-epi-lipoxin A4 via eNOS and iNOS induces NO synthesis, which mediates the anti-inflammatory effects of aspirin by negatively regulating leukocyte–endothelial interaction [98].

Statins, such as atorvastatin, also have the ability to enhance 15-epi-LXA4 formation. It is known that 15-epi-LXA4 is formed from 15R-HETE by the action of 5-LOX. By regulating and activating COX-2 and 5-LOX, statins can demonstrate anti-inflammatory and anti-atherosclerotic actions [95]. In addition, lovastatin increased the levels of 14,15-EET via CYP450, which increased the production of 15-epi-LXA4, which, however, has been shown in airway mucosa and requires additional research [99]. Moreover, the phosphorylation of 5-LOX at Ser^523^ by protein kinase A (PKA), which is induced by atorvastatin and pioglitazone, determines the formation of the anti-inflammatory 15-epi-LXA4 or pro-inflammatory LTB4 [100]. Atorvastatin and pioglitazone have been shown to increase 5-LOX levels in the cytosolic fraction [100]. This is because phosphorylation at Ser^271^ can promote [82,101], and phosphorylation at Ser^523^ by protein kinase A inhibits the nuclear import of 5-LOX [71,102].

Lipoxins and epi-lipoxins exert their action through the lipoxin A4 receptor/formyl peptide receptor 2 (ALX/FPR2, also called ALX receptor, FPR2 receptor, ALX/FPR, and FPRL1) [103]. The ALX/FPR2 mRNA levels were shown to be significantly elevated in atherosclerotic lesions compared with control healthy vessels. Moreover, in the region of human atherosclerotic lesions, ALX/FPR2 was expressed primarily on macrophages, as well as on VSMCs and endothelial cells [104]. The results suggest a dual role of ALX/FPR2 signaling in atherosclerosis. It is to promote disease progression by increasing the size of the atherosclerotic lesion, but atherosclerosis is characterized by a more stable plaque phenotype [105]. This is consistent with evidence that ALX/FPR2 promotes pro-inflammatory signaling in leukocytes, leading to accelerated atherosclerosis, while ALX/FPR2 expression in VSMCs potentially increased plaque stability [105]. Notably, in addition to transducing the anti-inflammatory effects of LXA4, the ALX/FPR2 receptor may also mediate the pro-inflammatory effects of serum amyloid A (SAA) and several other peptides [106,107,108,109,110].

In addition to ALX/FPR2, lipoxin A4 is considered as a ligand of the aryl hydrocarbon receptor (AhR) [111]. ATLs also bind to the CysLT1 receptor (Cysteinyl leukotriene receptor 1), while competing with leukotriene LTD4 [112]. A higher expression of the CysLT1 receptor has been reported in human carotid atherosclerotic lesions. This is consistent with evidence that the pro-inflammatory environment in atherosclerosis contributes to increased CysLT1 receptor expression through the stimulation of VSMCs [113,114]. For example, LPS stimulation has been shown to induce CysLT1 receptor expression in human coronary artery VSMCs [114,115].

LXA4 and LXB4 are characterized by multiple anti-inflammatory effects. They contribute to the inhibition of neutrophil transendothelial migration stimulated by LTB4 [116]. Although neutrophils are rarely found in atherosclerotic plaques, they are actively involved in the pathogenesis of atherosclerosis by contributing to inflammation [117]. Neutrophils also contribute to the destabilization of atherosclerotic plaques [118]. In contrast to the fact that LTC4 and LTD4 increased the adhesion of PMN to the endothelium, partially stimulating the mobilization of P-selectin, lipoxins can weaken the P-selectin-mediated adhesion of PMN to endothelial cells [116]. Lipoxin A4 and 15-epi-lipoxin A4 have been shown to modulate the expression of adhesion molecules on human leukocytes in whole blood and to inhibit neutrophil adhesion to endothelial cells [119]. Lipoxin A4 and lipoxin B4 inhibit neutrophil chemotactic responses stimulated by leukotriene B4 and N-formyl-L-methionyl-L-leucyl-L-phenylalanine [120]. In addition, LXA4, 15-epi-LXA4, and their synthetic analogues selectively reduce azurophilic PMN degranulation [121].

It should be noted that lipoxins have different effects on PMN and monocytes [122]. In contrast to the described effect on PMN, LXA4 and LXB4 stimulate monocyte chemotaxis and adhesion, which may play a role in physiological monocyte movement and/or pathological processes [122]. In addition, lipoxins increase the uptake of apoptotic neutrophils by macrophages [123], which promotes the clearance of apoptotic leukocytes by macrophages at the site of inflammation [123,124,125].

LXA4 and 15-epi-LXA4 also inhibit peroxynitrite formation, nuclear factor-κB (NF-kB) and AP-1 (activator protein-1) activation, and *IL-8* gene expression in leukocytes [126]. In addition, ATLs can impair angiogenesis by inhibiting endothelial cell proliferation and migration [127].

Recent studies convincingly show that VSMCs are actively involved in the pathogenesis of atherosclerosis [128]. They are the main cell type that is present at all stages of atherosclerotic plaque development. VSMCs exhibit phenotypic plasticity [129]. The cells derived from VSMCs are the main source of atherosclerotic plaque cells and the extracellular matrix [129]. VSMCs have been shown to have specific receptors for ATLs and resolvin E1, such as ALX/FPR2 and ChemR23. Because of this, ATLs and resolvin E1 can act on VSMCs to provide a protective phenotypic switch for these cells and may thus have further potential to prevent atherosclerosis [97]. In particular, the lipid mediators ATLs and RvE1 have been shown to be involved in counteracting the regulation of PDGF-stimulated VSMC chemotaxis [97]. In another study, the 15-epi-lipoxin A4 signals through ALX/FPR2 in vascular smooth muscle cells and protects against intimal hyperplasia after carotid artery ligation [105].

In a study evaluating the effect of lipoxin A4 on myocardial ischemia-reperfusion injury following cardiac arrest in a rabbit model, the inhibitory effect of LXA4 on NF-κB, IL-1β, IL-6, and TNF-α expression, as well as the infarct ratio and apoptotic index values, was shown. Another positive role was the improvement of the IL-10 expression, hemodynamic indices, and myocardial structure and function [130].

The regulation of reverse cholesterol transport is an important mechanism, the disruption of which is closely related to the formation of froth cells in the vascular wall. This process involves the active participation of ABCA1, a member of a large family of ABC transporters. ABCA1 regulates the reverse transport of cholesterol to extracellular acceptors, thereby regulating cholesterol accumulation in macrophages, and through this mechanism may be related to the involvement of these cells in inflammation. Interestingly, LXA4 can induce a dose-dependent increase in ABCA1 and LXRa expression and through this mechanism may be involved in the regulation of reverse cholesterol transport in THP-1 macrophage-derived foam cells [131]. These findings significantly broaden the view on the function of LXA4 in inflammation, as the decreased expression and functional activity of ABCA1 leads to impaired reverse cholesterol transport in macrophages and their inflammatory activation. Thus, the anti-inflammatory role of LXA4 mediated by increased ABCA1 expression represents an important antiatherogenic mechanism.

The involvement of lipoxins in cholesterol metabolism may also be mediated through the increased expression of another representative of the ABC transporters, Abcb11. Abcb11 provides lipid homeostasis through regulation of biliary lipid secretion. Lipoxins cause an increase in Abcb11 expression through a posttranscriptional and posttranslational mechanism involving MAPK p38 activity [132].

Thus, lipoxins are involved in the regulation of many pathophysiological mechanisms that are associated with the development of atherosclerosis. Recent evidence of the deficient production of 15-epi-LXA4 in patients with peripheral arterial disease suggests a protective role for lipoxins in atherogenesis [97]. Despite higher levels of circulating LXA4 in patients with coronary heart disease, lower local levels of LXA4 were observed in rabbit atherosclerotic vessel walls. It was also found that LXA4 inhibited oxLDL-induced regulation of CD36 and reduced oxLDL-induced macrophage apoptosis and foam cell formation [133].

It has also been shown that decreased serum LXA4 levels correlate with the development of metabolic syndrome [134]. In this regard, the assessment of LXA4 levels can be used for the early detection and prevention of metabolic syndrome.

Thus, lipoxins are considered to be an important tool that provides control of inflammation and regulation of inflammation resolution. The role of lipoxins in the prevention of atherosclerosis is a subject for study in order to find new drugs that could increase the effectiveness of treatment.

### 3.2. Resolvins

**Figure 4:**
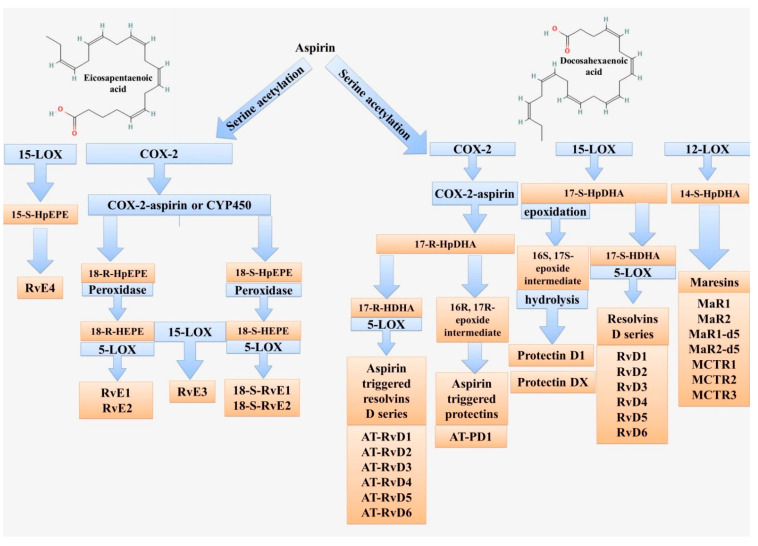
Scheme of lipid mediator biosynthesis from eicosapentaenoic and docosahexaenoic acids. These fatty acids are used to form resolvins (Rvs), maresins (MaRs), and protectins (PDs). The key enzymes are 5-LOX, 12-LOX, 15-LOX, and COX-2.

Resolvins (Rvs) are a family of bioactive derivatives of eicosapentaenoic acid (EPA) and docosahexaenoic acid (DHA) (Figure 4). Their synthesis from docosapentaenoic acid (DPA) and clupandonic acid (cis-7,10,13,16,19-Docosapentaenoic acid) has also been described [135]. Resolvins are small lipid molecules with anti-inflammatory and immunoregulatory properties [136,137]. The term “resolvins” is related to their function (short for resolution phase interaction products) and was first used to describe this group of substances [138,139]. Thus, Rvs have both pro-resolution anti-inflammatory and immunoregulatory properties. There are many studies that have shown the potential beneficial effects of resolvins on the course of various inflammatory processes and the therapeutic value from the use of this group of substances [135,140,141,142,143,144].

Among resolvins, there are the D-series resolvins (RvD) and the E-series resolvins (RvE) [136,145]. The main difference and the reason for the division into groups is the initial product for the synthesis and the slight differences in the chemical structure. RvD is biosynthesized from DHA, RvE is from EPA [146,147].

Currently, six representatives of RvD are known: RvD1 to RvD6. The key enzymes for their synthesis are LOX-15, LOX-5, and for some subtypes, such as aspirin-triggered RvD (AT-RvD), acetylated cyclooxygenase-2 (COX-2) and cytochrome P450 [148,149].

There is information in the literature about the possibility of using RvD1 for the therapy of certain diseases, such as Parkinson’s disease and Alzheimer’s disease [150,151], the prevention of proarrhythmic atrial remodeling [152], and others [153,154,155,156,157,158]. The mechanism of the anti-inflammatory action of RvD1 includes a stimulatory effect on ALX/FPR2 [159]. ALX/FPR2 is a G-protein-coupled receptor (GPCR) and can, depending on the ligand, exert both pro-inflammatory (agonists: N-formyl-Met-Leu-Phe-Lys (fMLFK), amyloidogenic proteins and antibacterial peptides) [160,161] and anti-inflammatory effects (agonist—RvD1, AT-RvD1, RvD3, LXA4, ATLs, Annexin A1) [161,162]. The ALX/FPR2 receptor is expressed on macrophages, endothelial cells, and smooth muscle cells, among others [105]. The effect of RvD1 on ALX/FPR2 alters the intracellular Ca^2+^ content through CaMKII and the subsequent inhibition of MAPK p38 phosphorylation [83,161,163,164]. The antioxidant effect of RvD1 is realized by reducing the activation of NF-κB and increasing the synthesis of antioxidant compounds, thereby reducing the formation of reactive oxygen species (ROS).

A number of studies have established a protective or neutral effect of the ALX/FPR2 receptor in macrophages on the development of atherosclerotic lesions [105]. This indicates the possibility of using the ALX/FPR2 receptor as a therapeutic target in atherosclerosis. However, one should not forget that the effects of ALX/FPR2 stimulation can be the opposite depending on the ligand.

In addition to their effects on ALX/FPR2, RvD1, other resolvins (RvD3, RvD5), and synthetic analogues of BDA-RvD1 (benzo-diacetylenic-17R-RvD1-methyl ester) act on DRV1 (the receptor is also known as GPR32). DRV1 is widely expressed in monocytes and macrophages and is also present in neutrophils and lymphocytes and on the membrane of cardiomyocytes [159,161,165,166,167]. In contrast to ALX/FPR2, the DRV1 receptor lacks murine homologues, which significantly complicates the in vivo study of the mechanisms of regulation by this receptor [115]. The main role of the receptor is to enhance phagocytosis, reduce PMN infiltration [168,169], and participate in the processes of inflammation resolution [61,170,171].

RvD1 has a regulatory effect on neutrophil migration through the endothelium [172,173], promotes neutrophil efferocytosis, and activates M2 type macrophages [174]. Other resolvins act similarly, affecting neutrophils and macrophages, and thereby exerting an anti-inflammatory effect [164,175]. In macrophages, RvD1 inhibits the release of pro-inflammatory cytokines such as IL-6 and TNF-α [139,176,177] and enhances the production of anti-inflammatory cytokines. The RvD1 levels have been shown to be reduced in individuals with carotid atherosclerosis [178,179]. A number of works by other researchers demonstrate the effect of RvD1 on atherosclerotic plaque stability; in addition, RvD1 contributes to the reduction in necroptotic cells (NCs). In addition, RvD1 activates the PI3K/Akt pathway, which reduces the negative effects of ischemia and reduces infarct size [179]. Thus, RvD1 may be a marker for the diagnosis of complications and a potential link in the therapy of atherosclerotic lesions [179,180,181]. RvD1 enhances necroptotic cell clearance by stimulating fatty acid oxidation and the oxidative phosphorylation of macrophages via AMPK signaling [181].

Of interest is the information that RvD1 regulates a number of microRNAs (miRNAs) targeting cytokines and some proteins involved in the immune system, which may contribute to the resolution of inflammation [170].

Another member of the resolvins, RvD2, has an important role in atherosclerotic lesions. RvD2, like RvD1, has anti-inflammatory properties in various pathological conditions [77,182], including vascular conditions [183]. This action is associated with the regulation of PMN infiltration, the effect on macrophages due to increased phagocytosis, and a decrease in the synthesis of PAF (platelet activating factor), LTB4, and PG [147]. RvD2 may be involved in the regulation of nitric oxide production. It has been shown to play a role in nitric oxide production as well as in the modulation of leukocyte adhesion receptor expression, which reduces the interaction between leukocytes and endothelium [184]. In addition, RvD2 promotes the release of prostacyclin from vascular endothelial cells [165,184]. All of these effects together indicate an important role for RvD2 in protecting the vascular wall from atherosclerotic lesions.

RvD3 has a specific role in the resolution of inflammation. It is synthesized in the late stages of inflammation resolution, characterized by the appearance and increased accumulation 24 h after the onset of inflammation. High levels persist for up to 72 h, which corresponds to the stage of inflammation resolution. RvD3, due to its high activity, helps to reduce PMN infiltration into tissues and the production of inflammatory mediators. In addition, it enhances the phagocytosis and efferocytosis of macrophages. 17R epimer (AT-RvD3), whose biosynthesis is triggered by aspirin, has similar properties [60,166].

RvD4 reduces the effects of deep vein thrombosis by regulating neutrophil infiltration and increasing monocyte levels and enhancing phagocytosis, whereas its metabolite, 17-oxo-RvD4, has virtually no involvement in phagocytosis and has no anti-inflammatory activity compared to RvD4 [185,186,187].

The therapeutic use of RvD1 in atherosclerosis contributes to plaque stabilization by reducing focal necrosis and oxidative stress; RvD2 and Mar1 have a similar effect in preventing atheroprogression [76,188]. In addition, the effects of RvD1 have been described as decreasing the number of neutrophils in the inflammatory zone and switching the macrophage phenotype toward M2 in the spleen and left ventricle [189,190]. However, RvD1 is chemically a complex molecule that is difficult to synthesize; so, more simply organized compounds with the ability to activate FPR2 or DRV1/GPR32 receptors have gained an advantage [191].

The resolvins E series (RvEs) includes resolvin E1 (RvE1), resolvin E2 (RvE2), resolvin E3 (RvE3), and resolvin E4 (RvE4) [192]. The substrate for their synthesis is eicosapentaenoic acid (EPA) [50,193]. The key enzymes of synthesis are endothelial aspirin acetylated COX-2, CYP450, and leukocyte 5-LOX (for RvE1 and RvE2) and 15-LOX (for RvE3 and RvE4) [194,195,196].

RvE1 and RvE2 are the most widely described and are the main representatives of the E-series resolvin family. RvEs exert their anti-inflammatory effects through their action on the E-series resolvin receptors (ERV), also known as chemokine-like receptor 1 (CMKLR1) or chemerin receptor 23 (ChemR23) [197]. ERV is expressed in neutrophils, monocytes, macrophages, and dendritic cells [198,199,200,201,202,203,204,205,206]. 

RvE1 has anti-inflammatory and pro-resolving effects through several mechanisms, in particular by reducing neutrophil migration [194,200,207,208,209,210], increasing the activation of the efferocytosis process of apoptotic neutrophils by macrophages [208,209], inhibiting the release of inflammatory mediators [211,212], and by regulating the monocyte-macrophage system [161,213,214]. In addition, RvE1 increases the expression of C-C chemokine receptor type 5 (CCR5), which also demonstrates the involvement of RvE1 in resolving inflammation [215].

RvE1 administration to ApoE*3-Leiden transgenic mice significantly reduces interferon gamma (IFN-γ), disintegrin, and metalloproteinase domain-containing protein 17 (ADAM17) and TNF-α, which are directly involved in atherogenesis. This occurs by regulating the expression of the genes encoding them. Moreover, against the background of ADAM17 reduction, the process of efferocytosis is activated and inflammation signaling is inhibited as ADAM17 influences MerTK, which in turn regulates efferocytosis and inflammation resolution in vivo [18,216]. In experimental animal models, RvE1 administration has been shown to suppress atherogenesis and vascular inflammation, which is an interesting subject for study in terms of new approaches to preventing atherogenic complications [217]. RvE1 reduced the area and severity of atherosclerotic lesions in experimental animals, favoring RvE1’s effect on the risk of plaque rupture [218]. 

RvE2 is another member of the RvEs family. Its chemical structure is very similar to RvE1 [149,207]. Resolvin RvE2 is known to be a substance with potent anti-inflammatory properties that inhibits zymosan-induced PMN infiltration in experimental peritonitis in mice. RvE2 is present in the blood plasma of healthy people [197] and is synthesized by PMN in significant amounts [149,207]. At concentrations of 1–10 nM, it has a direct regulatory effect on human neutrophil chemotaxis processes and promotes the activation of phagocytosis and the production of anti-inflammatory cytokines. In addition, it prevents platelet aggregation, indicating its protective properties and role as a local mediator of tissue homeostasis during inflammation resolution [197,219].

RvE3 and RvE4 are less studied compared to the aforementioned E-series resolvins. They are synthesized mainly by neutrophils and macrophages under hypoxia and have the ability to inhibit neutrophil migration and stimulate the efferocytosis of senescent red blood cells (SRBC) and apoptotic neutrophils by M2 macrophages [146,192,220]. This is confirmed in experiments on human cells and in vivo experiments in mouse models of inflammation [146,147,220]. Moreover, resolvin RvE3 in experiments suppresses the process of the chemotaxis of polymorphonuclear leukocytes [192].

Pathways of therapeutic action on ERV1/ChemR23 receptors can be used to reduce inflammation in cardiovascular diseases, such as atherosclerotic vascular lesions. RvE1 is known to have anti-inflammatory effects in vessels and to attenuate atherosclerotic vascular lesions both in monotherapy and in combination with statins without affecting cholesterol levels and lipid spectrum [218]. Influencing vascular inflammation with inflammation resolution mediators represents a new approach to preventing atherosclerotic vascular lesions [217].

Analysis of the above data points to the important role of D-series and E-series resolvins in the mechanisms of inflammation resolution. Their dysregulation may be part of the pathogenesis of many inflammatory diseases, including atherosclerosis.

### 3.3. Protectins

Protectins (PDs) are other members of the SPM family that can be formed from the two omega-3 PUFAs, docosahexaenoic acid (DHA) and docosapentaenoic acid (DPA) [221]. PDs have three conjugated double bonds located between the 10th and 17th carbon atoms and are chemically E,E,Z-docosatrienes. The best-known member of this SPM group, Protectin D1 (PD1 or 10R,17S-dihydroxy-docosa-4Z,7Z,11E,13E,15Z,19Z- hexaenoic acid), is a dihydroxylated noncyclic docosatriene, which is formed by lipoxygenation and hydrolysis of an epoxy intermediate (Figure 4).

The first descriptions of PD1 linked its action to protection against oxidative stress in brain and retinal tissues; so, it was named Neuroprotectin D1 (NPD1) [222,223]. Subsequent studies have expanded the understanding of its functions in different tissues. PD1 is known to be produced by PMNs [224], macrophages [225], and eosinophils [226,227].

Protectins exert their anti-inflammatory effect through a special kind of G-protein-coupled receptor, GPR37 (G-protein-coupled receptor 37) or PAELR (parkin-associated endothelin receptor-like receptor) [221]. The strength of the anti-inflammatory effect of protectins is related to the stereochemistry of the molecules. For example, the R-epimer of PD1 has greater activity than the S-epimer [221,228].

PD1 exhibits potent anti-apoptotic and anti-inflammatory activity. The anti-inflammatory effects of PD1 include the inhibition of neutrophil migration [229] and the reduction in TNF-α and IFN-γ production by neutrophils [230]. In addition, PD1 regulates CCR5 expression in neutrophils [215] and stimulates macrophage phagocytosis and efferocytosis [58,166,231]. PD1 contributes to the reduction in neutrophil infiltration of tissues and increases the phagocytic activity of macrophages to engulf apoptotic neutrophils [208]. This is an important part of the mechanism of inflammation resolution and may be part of the early anti-inflammatory response in CHD. An increase in the level of protectins in the first hours after myocardial infarction, with a subsequent decrease to normal values, has been shown. Moreover, the PD2n-3 DPA and PD1 levels were positively correlated with the number of neutrophils after the onset of myocardial infarction. These data suggested the involvement of protectins as a counteracting mechanism to attenuate the negative effect of the initial neutrophil increase after ST-elevation myocardial infarction (STEMI) [14].

At present, in addition to PD1, protectin DX (PDX or 10S,17S-dihydroxy-4Z,7Z,11E,13Z,15E,19Z-docosahexaenoic acid) is well known. PDX is a geometric stereoisomer of PD1. PD1 and PDX differ in the geometry of the double bonds in the conjugated triene, which is E,Z,E for PDX and E,E,Z for PD1, as well as in the configuration of the carbon 10, which is S in PDX and R in PD1. Despite the similarity in chemical structure, the biological properties of PD1 and PDX are different [232]. Moreover, the biological activity attributed to PD1 may be related to its PDX isomer [232]. PDX inhibits COX-1 and COX-2, thereby reducing the formation of pro-inflammatory prostaglandins [233]. Unlike PD1, PDX inhibits platelet aggregation by inhibiting COX-1. PDX also inhibits TxA2-induced platelet aggregation [232,234]. Both the formation and the action of endogenously formed thromboxane can be a target for PDX [233]. PDX also reduces the production of ROS and COX activity and the release of myeloperoxidase from neutrophils [232,235]. However, PDX has no effect on the 5-LOX pathway, which produces LTB4 [235].

PD1 metabolic products such as 22-OH-PD1 (22-hydroxy-PD1 or 10R,17S,22-trihydroxy-4Z,7Z,11E,13E,15Z,19Z-docosahexaenoic acid), which also exhibit potent anti-inflammatory activity [236], are also of interest. 22-OH-PD1 is an omega-oxidation product of PD1 and contributes to the inhibition of PMN chemotaxis. Aspirin-triggered PD1 (AT-PD1 or 17-epi-PD1, i.e., 10R,17R-dihydroxy-4Z,7Z,11E,13E,15Z,19Z-docosahexaenoic acid) is a potent anti-inflammatory molecule that is formed with aspirin. AT-PD1 contributes to the resolution of inflammation by reducing the transendothelial migration of PMNs, as well as by enhancing the efferocytosis of apoptotic PMNs by macrophages [228]. 

Thus, the role of protectins in the resolution of inflammation and in the pathogenesis of atherosclerosis is of clinical interest and requires further research [138]. 

### 3.4. Maresins

Maresins (MaRs), other SPMs, are derived from ω-3 docosahexaenoic acid (DHA) [237]. They are produced by macrophages, for which they received their name (MAcrophage, RESolving INflammation) [237]. Several members of this class have been described: MaR1, MaR2, MaR1-d5, MaR2-d5, MCTR1, MCTR2, MCTR3, of which the last three maresins are conjugated substances (maresin conjugate in tissue regeneration, MCTR) [238]. Their chemical structures are close to each other, which explains their common functions and the mechanism of anti-inflammatory action, but at the same time, maresins differ in their activity and specificity [239]. In addition, there is evidence for maresin-like (L) mediators, such as maresin-L1 and maresin-L2, which are enantiomers of each other. They, like the true maresins, have anti-inflammatory and reparative effects but are produced by both activated macrophages and leukocytes and platelets, with maresin-L1 being produced 10 times more than its enantiomer, maresin-L2 [240,241].

Maresin biosynthesis starts with a substrate, which is DHA (Figure 4). The key enzyme of the synthesis is 12-LOX [242,243]. MaR1 was first described as a product of DHA conversion by macrophages derived from human monocytes [237] and is a dihydroxylated docosatriene isomer of PD1 [232]. However, unlike protectins, which can be produced by neutrophils, maresins are mainly produced by M2 macrophages and provide a potent anti-inflammatory effect. By providing resolution of inflammation, MaR1 has analgesic and regenerative effects, and an antiaggregant effect has also been found, indicating a protective role, including in vascular damage [147,244,245,246,247,248,249].

Maresins, being isomers, are capable of transferring into each other. Enzymes such as epoxide hydrolase [250], soluble epoxide hydrolase [251], leukotriene C4 synthase and glutathione S-transferase MU 4, gamma-glutamyltransferase, and dipeptidase [242,252,253], which catalyze the transition to one or another maresin, play a key role in this process.

The mechanism of the anti-inflammatory action of MaRs is to enhance the phagocytosis and efferocytosis of macrophages and to limit the penetration of polymorphonuclear leukocytes [188,249,251,254,255,256]. In addition, MaRs contribute to the reduction in inflammatory mediators such as IL-6, IL-1β, and TNF-α [77,257] and increase the production of anti-inflammatory mediators (IL-10) [242,258]. The biological role of MCTR conjugated maresins, which appear at a later stage, is to regulate the mechanisms of inflammation resolution and tissue regeneration [147,247,249].

Two receptors are targeted by MaR1: retinoic acid-related orphan receptor-α (RORα) and Leucine-rich repeat-containing G-protein-coupled receptor 6 (LGR6) [259,260]. Specific MaR1 binding enhanced efferocytosis and phagocytosis and also promoted phosphorylation of several proteins, including ERK and cAMP responsive element-binding protein (CREB1) [260]. The property of MaR1 to suppress oxidative stress, presumably through activation of the Nrf2-mediated HO-1 signaling pathway, has been described [261].

MaR1 directly enhances neutrophil activation and plays an important role in switching macrophages from the M1 to the M2 phenotype [248,249,262]. MaR1 mainly acts on vascular endothelial cells and VSMCs and promotes the reduction in TNF-α-induced monocyte adhesion to the endothelium. In addition, it has an antioxidant effect, which is manifested through its effect on the factor NF-κB [263]. When the levels of MaR1 and RvD2 decreased, the progression of atherosclerosis was observed, which may be associated with impaired efferocytosis against the background of the reduction in these mediators [188]. There is evidence that RvD2 and MaR1 prevented the progression of atherosclerosis by causing a change in the macrophage profile toward a reparative phenotype, which secondarily stimulated collagen synthesis in smooth muscle cells [188]. This indicates a homeostatic effect of MaR1 on vascular cells, which is of no small importance in acute and chronic vascular inflammation and can be used as a therapeutic target in vascular lesions [263].

MaR2 showed similar effects to MaR1, limiting PMN infiltration and enhancing phagocytosis, while being 2–3 times less active than MaR1 [239].

MCTR1, MCTR2, and MCTR3 maresin conjugates are involved in tissue regeneration and the regulation of PMN infiltration [238,261]. In a mouse model, MCTR1 has been shown to promote the accumulation of M2 macrophages and significantly accelerate the resolution of inflammation [264]. In addition, it contributes to a decrease in the production of inflammatory cytokines such as TNF-α, IL-1β, and IL-6. In addition, all MCTRs interact with CysLT1, reducing vascular permeability and affecting cardiac function [238]. 

Thus, MaRs provide a regulatory influence on the processes of inflammation resolution and are of interest in the pathogenesis of atherosclerosis [265,266].

## 4. Pharmacology of Inflammation Resolution in Atherosclerosis Involving Lipid Mediators

### 4.1. Medications Involved in the Regulation of SPM Biosynthesis

The evidence accumulated to date suggests that a number of medications can affect lipid mediator biosynthesis pathways and contribute to the regulation of inflammation. As previously noted, aspirin is involved in the production of SPMs. ATLs exhibit a longer half-life in vivo, which is probably due to the fact that it is a less efficient substrate for metabolizing enzymes because of its configuration [267,268]. Even low doses of aspirin have been shown to promote ATL production [269]. This pharmacological effect is of clinical interest given the antiplatelet activity of aspirin, which generally demonstrates a positive role for the drug in atherosclerosis.

Representatives of another group of medications that are widely used in cardiology practice, statins, also demonstrate a positive role in the production of SPMs. Statins have found widespread use in the treatment of atherosclerosis as effective hypolipidemic agents. This group of medications is known to have a wide range of pleiotropic effects, including participation in the regulation of inflammation through the production of ATLs. 

Of the other group of medications positively related to the formation of lipid mediators associated with the resolution of inflammation, thiazolidinedione derivatives should be noted. Rosiglitazone and pioglitazone are used as antidiabetic drugs to improve insulin resistance [270,271]. Pioglitazone and atorvastatin increase the expression and activity of cPLA2 and COX-2 in the mouse heart in an experiment. cPLA2 in turn releases arachidonic acid from cell membranes for its further metabolism into prostaglandins, leukotrienes, and lipoxins [95]. Thiazolidinediones also increase arachidonic acid release by inhibiting arachidonic acid reuptake [271]. High concentrations of rosiglitazone and pioglitazone significantly increased lipopolysaccharide-stimulated production of prostanoids such as TXA2 and PGE2 [271]. In addition, pioglitazone has been shown to increase plasma levels of 15-epi-LXA4 in patients with type 2 diabetes [272]. These data are of particular interest given the clinical significance of the course of atherosclerosis in diabetes mellitus. In addition, it has been shown that pioglitazone can stabilize coronary plaque. This is associated with a decrease in the necrotic core component combined with an increase in plasma adiponectin levels [273]. In an experimental model of stroke in rats, rosiglitazone was shown to induce 5-LOX expression, causing a switch from pro-inflammatory LTB4 synthesis to LXA4 synthesis [274].

Thus, a number of known drugs have new therapeutic potential through involvement in lipid mediator metabolism. This may be useful for further research to improve the efficacy of the treatment of atherosclerosis and its clinical consequences.

### 4.2. Clinical Perspectives on the Regulation of Inflammation Resolution

Given that atherosclerosis is a chronic progressive disease with an imbalance between inflammation activation and resolution, the pharmacological enhancement of inflammation resolution is a promising strategy. This new direction has been termed “resolution pharmacology” [275].

Despite the described positive molecular effects, the therapeutic potential of SPM is limited. This is because endogenously produced LXA4 and LXB4 are subject to rapid metabolic inactivation by prostaglandin dehydrogenase (PGHD) via dehydrogenation and ω-oxidation, which largely limits their clinical prospects [276]. This fact serves as the basis for the development of more stable synthetic analogues. The search for analogues is carried out along several lines based on the chemical structure of the lipoxins [276]. First-generation lipoxin analogs derived by carbon-15 and omega-end modification (15(R/S)-methyl-lipoxin A4 (ATLa1) and 15-epi-16-(para-fluoro)-phenoxy-LXA4 (ATLa2)) had limited therapeutic potential because of their rapid clearance [277,278]. Second-generation lipoxin analogs derived by incorporating the 3-oxa group (ZK-996, ZK-990, ZK-994, and ZK-142) had broad anti-inflammatory effects following intravenous, oral, and topical administration and had enhanced metabolic and chemical stability [279]. The 3-oxa-ATL analogues, which have a good pharmacokinetic profile, provide new opportunities to explore the therapeutic potential of LX and ATL [279]. Replacement of the triene core of LXA4 with a benzene ring increases metabolic stability while preserving useful biological activity [268,280,281]. In an experiment in ApoE-/- mice with diabetes, the administration of LXA4 and Benzo-LXA4 resulted in attenuation of aortic plaque development and inflammatory responses in aortic tissue, including expression of VCAM-1, MCP-1, IL-6, and IL-1β. In mice with atherosclerosis, treatment with benzo-LXA4 (1R)-3a for 6 weeks, initiated 10 weeks after the onset of diabetes, resulted in a significant reduction in plaque development in the aortic arch [282].

In addition to lipoxins, efforts on the pharmacological application of SPMs have focused on the search for synthetic analogues of resolvins. Several RvD1 analogs have been developed, including 17-(R/S)-methyl-RvD1 methyl ester and 17R-hydroxy-19-para-fluorophenoxy-RvD1 methyl ester, whose clinical applications have been evaluated in ophthalmology and in lung injury [278,283,284]. In addition, an analogue of RvD1 (benzo-diacetylenic-17R-RvD1-methyl ester (BDA-RvD1)) was developed, which, like endogenous RvD1, caused a decrease in neutrophil infiltration and the stimulation of phagocytosis [275,285]. A synthetic analogue of resolvin (Benzo-Rvd1) attenuates VSMC migration and neointimal hyperplasia [286].

The data on the prospects of nanoparticles containing aspirin-triggered resolvin D1 (AT-RvD1) or a stable analog of LXA4 seem interesting. These nanoparticles demonstrated anti-inflammatory properties in the experiment by significantly reducing neutrophil influx in a murine peritonitis model. In addition, the nanoparticles also reduced resolution intervals and showed pro-resolution actions accelerating keratinocyte healing. The enriched nanoparticles were also shown to protect against inflammation in the temporomandibular joint [287]. These results show that nano-pro-resolving medicines (NPRMs), which are mimetics of endogenous resolving mechanisms, have useful therapeutic properties [191,287].

Of clinical interest is the information about synthetic FPR2 agonists, which play the most promising role in “resolution pharmacology” [275,288]. A study of the properties and clinical effects of compound 43 (Cmpd43), which is a dual FPR1/FPR2 agonist, showed that Cmpd43 stimulated FPR1/2-mediated signaling, thereby enhancing pro-resolution cellular function and modulating cytokines [289]. The clinical effects of Cmpd43 are associated with improved left ventricular function and reduced left ventricular remodeling after myocardial infarction. This corresponds to an increase in pro-resolute macrophage markers. The findings suggest that FPR agonism improves cardiac structure and function after myocardial infarction [289]. Based on the data obtained in the Cmpd43 studies, Compound 17B (Cmpd17B) was synthesized, which is a pyridazinone derivative and demonstrates cardioprotective properties by influencing the ERK signaling pathway in both FPR receptors, which provides an effective anti-inflammatory effect during the acute phase of inflammation [189,290,291]. The selective FPR2 agonist BMS-986235/LAR-1219 inhibited neutrophil chemotaxis and stimulated macrophage phagocytosis, and in a mouse model of heart failure, it improved cardiac structure and functional performance [292]. It should be noted that receptor ligands can act in different ways on inflammation; so, this direction of pharmacological search should be strengthened by future studies.

It is of interest to know that chemerin-9, a potent agonist of the ChemR23 receptor, prevents atherogenesis in the experiment [293]. In addition, chemerin-9 has been shown to attenuate abdominal aortic aneurysm formation in ApoE-/- mice [294]. In addition to chemerin-9, other active molecules are known, such as chemerin-13 (C13), the vascular effects of which still need to be studied [295,296].

Given the important cross-talk role of 5-LOX in inflammation, its inhibitors are considered a promising therapeutic agent. VIA-2291, which is a potent 5-LOX inhibitor, has been shown to reduce leukotriene production 12 weeks after acute coronary syndrome. It was found to reduce uncalcified plaque volume after 24 weeks compared to a placebo [297]. In another study, VIA-2291 was shown to effectively reduce leukotriene production. However, the 5-LOX inhibition of VIA-2291 was not associated with a significant reduction in vascular inflammation or blood inflammatory markers [298]. The use of VIA-2291 resulted in slower plaque progression compared with a placebo for various plaque subtypes in patients with recent acute coronary syndrome in study NCT00358826 [299]. In addition, the effect of the 5-lipoxygenase inhibitor VIA-2291 (Atreleiton) on epicardial fat volume in patients with recent acute coronary syndrome has been shown [300]. This seems important given that epicardial adipose tissue is associated with coronary atherosclerosis and may be considered a predictor of adverse cardiovascular events. In addition, administration of the 5-LOX inhibitor Zileuton to subjects selected for leukotriene risk haplotypes reduces LTB4 levels while improving endothelial dysfunction in patients with coronary heart disease [301].

It should be noted that the current clinical experience with the inhibition of the 5-LOX pathway for the treatment of atherosclerosis is limited and does not allow definitive conclusions about the efficacy of this therapeutic approach [302]. In addition, there is no definitive understanding of how 5-LOX inhibition may be related to the resolution of inflammation, given its role in SPMs production.

Another avenue that efforts to find new drug therapies for atherosclerosis have also focused on is FLAP, whose role in the production of lipid mediators associated with inflammation is well known. These efforts are aimed at reducing the production of leukotrienes and reducing the activation of inflammation. It should be noted that several generations of FLAP modulators are known to date and have been evaluated for the treatment of diseases with an inflammatory component in the pathogenesis, such as asthma [303]. Attempts to find applications of FLAP inhibition in the treatment of cardiovascular diseases continue. 

A phase 2a study (FLAVOR) was conducted to evaluate the efficacy of AZD5718, which is a reversible FLAP inhibitor in patients with recent myocardial infarction [304]. The study showed good tolerability and dose-dependent inhibition of leukotriene biosynthesis in the absence of significant improvement in coronary microvascular function as measured by echocardiography [305,306]. Of note, another earlier FLAP inhibitor, AZD6642, has not been clinically evaluated because of cardiovascular safety concerns [302,307]. 

The FLAP antagonist BRP-201 causes a switch in the class of lipid mediators produced in human macrophages, shifting LT biosynthesis toward SPMs [92]. This may be due not only to FLAP inhibition but also to stimulation of 15-LOX-1 activity in M2 macrophages [92]. In turn, BRP-187 inhibits LT biosynthesis by preventing 5-LOX/FLAP interaction on the nuclear envelope of human leukocytes without blocking the nuclear redistribution of 5-LOX. In addition, BRP-187 also inhibited microsomal prostaglandin E2 synthase-1 [308]. It should be noted that 5-LOX and FLAP inhibitors block inflammation but can also inhibit its resolution.

Thus, inhibition of 5-LOX and FLAP is considered a promising strategy for treating some diseases with an inflammatory component in the pathogenesis. However, there are still insufficient studies that can demonstrate the full range of effects of such inhibition on lipid mediator production and its consequences on inflammation activation and resolution. 

In this regard, to a greater extent, the drugs from these groups are used in the treatment of asthma, the pathogenesis of which is characterized by the participation of lipid mediators of inflammation [309,310]. 

Thus, the therapeutic potential of SPMs in the treatment of atherosclerosis remains untapped to date. The complexity of the cross-linkages that are involved in the biosynthesis of pro- and anti-inflammatory mediators, the high lability of these processes, and the intercellular nature of SPM production are subjects for further research, which should help define new directions in the “resolution pharmacology” of inflammation (Table 1).

## 5. Conclusions

A growing body of evidence confirms that the pathogenesis of atherosclerosis is closely related to lipids, from their accumulation in the arterial intima to their involvement in inflammation. Lipid mediators, which are derived from PUFAs, show complex roles in inflammation. They are involved in the initiation, maintenance, and resolution of inflammation. Regulation of these processes and maintenance of the balance between protective function and tissue damage are impaired in atherosclerosis. SPMs are physiological tools that are used by the innate immune system to regulate inflammation. Disruption of SPM formation or impaired coordination between lipid mediators involved in inflammation initiation and resolution is considered to be an important pathogenetic mechanism of atherogenesis. The regulation of SPM formation and function is viewed as a promising therapeutic target. Aspirin and statins may play a significant role in resolving inflammation through their involvement in the production of SPMs, which is an important therapeutic effect of these medications and should be the subject of new research.

The pharmacological potential of SPMs and their synthetic analogues in the treatment of atherosclerosis is currently limited. This is due to the complexity of cross-linkages between the pathways of the biosynthesis of inflammatory mediators, the mediators of inflammation resolution, and the not fully understood involvement of different cells involved in the formation of SPMs. At the same time, it should be noted that the search for solutions to these issues is a promising area of pharmacological search.

Promising directions for future studies may be a detailed analysis of intercellular interactions in the biosynthesis of SPMs, as well as the study of the disorders of these interactions in atherosclerosis. In addition, the study of crosslinks in the mechanisms regulating the initiation and resolution of inflammation should be further developed. It also seems important to continue studying the receptor signaling pathways through which SPMs exert their pro-resolving effects. It also seems important to understand how effects on key enzymes in the biosynthesis of lipid mediators of inflammation, such as 5-LOX or FLAT, may affect SPM production. These findings, as well as information on the mechanisms that link inflammation in the vascular wall, hemodynamic blood flow characteristics, dyslipidemia, and other risk factors, may further enhance understanding of the directions for finding new drugs to treat atherosclerosis. Future studies of synthetic analogues of SPMs and technologies for their delivery to the lesion site in the vascular wall are also of considerable interest. 

Thus, atherosclerosis is a chronic multifactorial disease, the development and progression of which are associated with an imbalance between pro- and anti-inflammatory factors. A better understanding of the processes that involve lipid mediators will allow us to find the keys to the problem of effective treatment of atherosclerosis and its complications.

## Figures and Tables

**Table 1:** Clinical perspectives on the regulation of inflammation resolution.

Group	Expected Beneficial Effect	Members	Prospects for Use in Atherosclerosis. Data on the Results of Research (ClinicalTrials.Gov Identifier)	Reference
PUFAs	Precursors for the biosynthesis of SPMs; other beneficial effects.	ω-3 PUFAs	Numerous clinical trials have been conducted (NCT01310270, NCT00764010, NCT01367145, etc.). Additional research is required.	[68,311,312]
Medications involved in the biosynthesis of SPMs	Production of aspirin-triggered SPMs; other beneficial clinical effects (antiplatelet activity; hypolipidemic effect; improvement of insulin resistance)	- Aspirin - Statins - Pioglitazone	Aspirin and statins have proven efficacy in treating patients with atherosclerosis. Additional research is required.	[95,268,269,272,274,313]
Synthetic analogues of SPMs	Anti-inflammatory effects; Pro-resolving actions;	Lipoxin analogues (4 generation)	It is the subject of studies on efficacy and safety. Many issues about pharmacodynamics and pharmacokinetics are unresolved.	[276,277,278] [285,286]
	Anti-inflammatory effect; attenuation of neutrophil infiltration and stimulation of phagocytosis; attenuation of VSMC migration and neointimal hyperplasia	Resolvin analogues	It is the subject of studies on efficacy and safety. Many issues about pharmacodynamics and pharmacokinetics are unresolved.	
Synthetic FPR2 agonists	Anti-inflammatory effects; pro-resolving action; cardioprotective properties	Compound 43 (Cmpd43) Compound 17B	Cmpd43 and Cmpd17b have shown positive effects in preclinical trials.	[189,291,292,314,315,316]
		BMS-986235/ LAR-1219	BMS-986235 demonstrated positive effects in preclinical trials. BMS-986235 is in phase I clinical trials (NCT03335553)	
		ACT-389949	ACT-389949 is in phase I clinical trials (NCT02099071, NCT02099201). No data on studies to evaluate efficacy in atherosclerosis. Additional research is required.	
5-LOX inhibitors	Reduces the production of leukotriene	VIA-2291/ Atreleuton/ Abbott-85761	Atreleuton had been in phase II clinical trials for the treatment of acute coronary syndrome and atherosclerosis (NCT00352417, NCT00358826, NCT00552188). The research was discontinued	[297,298,299,300,301,317,318]
		Zileuton	Studied in patients with asthma and COPD; there are studies in coronary heart disease.	
		Setileuton/ MK 0633	Setileuton has been in phase II clinical trials for the treatment of atherosclerosis (NCT00421278). Further clinical trial discontinued	
FLAP inhibitors	Inhibition of leukotriene biosynthesis	AZD5718/ Atuliflapon	Phase II clinical trial in patients with acute coronary syndrome (NCT04601467)	[302,305,308,319,320,321,322,323,324,325]
	Induces a switch in the formation of pro-inflammatory 5-LOX derivative LT towards inflammation-resolving 12/15-LOX derivative SPMs	BRP-201	No data on studies to evaluate efficacy in atherosclerosis	
	Inhibited LT biosynthesis; inhibited microsomal prostaglandin E2 synthase-1	BRP-187	No data on studies to evaluate efficacy in atherosclerosis	
	Inhibition of leukotriene biosynthesis	BAY X 1005/ veliflapon	Studied in patients with acute coronary syndrome (Phase 3, NCT00353067). Participant enrollment has been suspended.	

## Data Availability

Not applicable.

## Abbreviations

ABCA1	ATP binding cassette subfamily A member 1
ABCG1	ATP binding cassette subfamily G member 1
ADAM17	disintegrin and metalloproteinase domain-containing protein 17
AhR	aryl hydrocarbon receptor
ALX/FPR2	lipoxin A4 receptor/formyl peptide receptor 2
AMPK	AMP-activated protein kinase
AP-1	activator protein-1
ATLs	aspirin triggered lipoxins
AT-PD1	aspirin-triggered PD1
AT-RvD1	aspirin-triggered resolvin D1
BDA-RvD1	benzo-diacetylenic-17R-RvD1-methyl ester
CaMKII	Ca^2+^/calmodulin-dependent protein kinase II
CCR5	C–C chemokine receptor type 5
ChemR23	chemerin receptor 23
CMKLR1	chemokine-like receptor 1
Cmpd43	compound 43
COX	cyclooxygenase
CREB1	cAMP responsive element-binding protein 1
CysLT1 receptor	cysteinyl leukotriene receptor 1
DHA	docosahexaenoic acid
DRV1	resolvin D1 receptor
EETs	epoxyeicosatrienoic acids
eNOS	endothelial nitric oxide synthase
EPA	eicosapentaenoic acid
ERK	extracellular-signal-regulated kinase
FLAP	five lipoxygenase activating protein
fMLFK	N-formyl-Met-Leu-Phe-Lys
GPCR	G-protein-coupled receptor
GPR37	G-protein-coupled receptor 37
HETE	hydroxyeicosatetraenoic acid
HO-1	heme oxygenase 1
HpETE	hydroperoxyeicosatetraenoic acid
IFN	interferon
IL-4	interleukin-4
iNOS	inducible nitric oxide synthase
LDL	low-density lipoprotein
LGR6	leucine-rich repeat-containing G-protein-coupled receptor 6
LOX	lipoxygenase
LPS	lipopolysaccharide
LT	leukotriene
LX	lipoxin
MaR	maresin
MCP-1	monocyte chemoattractant protein 1
MCTR	maresin conjugate in tissue regeneration
MerTK	myeloid-epithelial-reproductive tyrosine kinase
MK2	MAPK-activated protein kinase 2
NCs	necroptotic cells
NF-kB	nuclear factor-κB
NPD1	neuroprotectin D1
Nrf2	nuclear factor erythroid 2-related factor 2
PAELR	parkin-associated endothelin receptor-like receptor
PAF	platelet activating factor
PD1	protectin D1
PDX	protectin DX
PG	prostaglandin
PI3K/Akt	phosphatidylinositol–3–kinase and protein kinase B
PMN	polymorphonuclear neutrophils
PUFAs	polyunsaturated fatty acids
RORα	retinoic acid-related orphan receptor–α
ROS	reactive oxygen species
RvD	resolvin D
RvE	resolvin E
SERCA2	sarcoplasmic/endoplasmic reticulum calcium ATPase 2
SPMs	specialized pro–resolving mediators
SRBC	senescent red blood cells
STEMI	ST-elevation myocardial infarction
TLR	toll-like receptor
TNF-α	tumor necrosis factor alpha
VCAM-1	vascular cell adhesion molecule 1
VSMCs	vascular smooth muscle cells
